# Lymphatic Dissemination of Simian Immunodeficiency Virus after Penile Inoculation

**DOI:** 10.1128/JVI.02947-15

**Published:** 2016-03-28

**Authors:** Zhong-Min Ma, Joseph Dutra, Linda Fritts, Christopher J. Miller

**Affiliations:** Center for Comparative Medicine and California National Primate Research Center, University of California, Davis, Davis, California, USA

## Abstract

The human immunodeficiency virus (HIV) is primarily transmitted by heterosexual contact, and approximately equal numbers of men and women worldwide are infected with the virus. Understanding the biology of HIV acquisition and dissemination in men exposed to the virus by insertive penile intercourse is likely to help with the rational design of vaccines that can limit or prevent HIV transmission. To characterize the target cells and dissemination pathways involved in establishing systemic simian immunodeficiency virus (SIV) infection, we necropsied male rhesus macaques at 1, 3, 7, and 14 days after penile SIV inoculation and quantified the levels of unspliced SIV RNA and spliced SIV RNA in tissue lysates and the number of SIV RNA-positive cells in tissue sections. We found that penile (glans, foreskin, coronal sulcus) T cells and, to a lesser extent, macrophages and dendritic cells are primary targets of infection and that SIV rapidly reaches the regional lymph nodes. At 7 days after inoculation, SIV had disseminated to the blood, systemic lymph nodes, and mucosal lymphoid tissues. Further, at 7 days postinoculation (p.i.), spliced SIV RNA levels were the highest in the genital lymph nodes, indicating that this is the site where the infection is initially amplified. By 14 days p.i., spliced SIV RNA levels were high in all tissues, but they were the highest in the gastrointestinal tract, indicating that the primary site of virus replication had shifted from the genital lymph nodes to the gut. The stepwise pattern of virus replication and dissemination described here suggests that vaccine-elicited immune responses in the genital lymph nodes could help prevent infection after penile SIV challenge.

**IMPORTANCE** To be the most effective, vaccines should produce antiviral immune responses in the anatomic sites of virus replication. Thus, understanding the path taken by HIV from the mucosal surfaces, which are the site of virus exposure, to the deeper tissues where the virus replicates will provide insight into where AIDS vaccines should produce immunity to be the most effective. In this study, we determined that, by day 7 after penile inoculation, SIV has moved first to the inguinal lymph nodes and replicates to high levels. Although the virus is widely disseminated to other tissues by day 7, replication is largely limited to the inguinal lymph nodes. The step-by-step movement of SIV from penile mucosal surfaces to the draining lymph nodes may allow an HIV vaccine that produces immunity in these lymph nodes to block HIV from establishing an infection in an exposed person.

## INTRODUCTION

The human immunodeficiency virus (HIV) is primarily transmitted by heterosexual contact, and approximately equal numbers of men and women worldwide are infected with the virus (1). Understanding the biology of HIV acquisition and dissemination in men exposed to the virus by insertive penile intercourse is likely to help with the rational design of vaccines that can limit or prevent HIV transmission. The need to understand the biology of penile HIV transmission was made clear after the phase IIb STEP trial of the MRKAd5/HIV-1 gag/pol/nef vaccine. This vaccine induced HIV-specific T cells that were associated with reduced HIV RNA levels in the plasma of some people; however, the vaccine also increased HIV acquisition in adenovirus serotype 5 (Ad5)-seropositive men with intact foreskins (2). Determining how Ad5 infection and subsequent immunization with the MRKAd5/HIV-1 gag/pol/nef vaccine enhanced penile HIV transmission is critical to the development of safe and effective HIV vaccines. Understanding the target cells and dissemination pathways involved in penile HIV transmission will greatly facilitate that goal.

The current study focuses on understanding penile HIV transmission in uncircumcised men, as approximately 70% of the male population worldwide is not circumcised (3) and because the enhanced HIV acquisition in the STEP trial was seen only in men with intact foreskins. Further, circumcision decreases the rate of HIV infection in heterosexual men by only 50 to 60% (4–8), indicating that other regions of the penis also play an important role in HIV transmission. The proximal portion of the penis (shaft) is covered by a dry keratinized squamous epithelium that is relatively resistant to HIV infection, unless the skin is broken, inflamed, or infected with a sexually transmitted pathogen. The distal potion of the penis is comprised of the glans, coronal sulcus, and the fold of skin covering the urethral meatus and glans of the penis, termed the foreskin or prepuce (9). The partially keratinized stratified squamous epithelium covering the surfaces of the subpreputial cavity, the glans and inner foreskin, is thinner and less cornified than the outer foreskin epithelium (9). The glans and inner foreskin tend to be moist (9, 10), although the glandular structures that typically produce mucosal secretions are not present in the lamina propria (11). Of note, sebaceous glands, normal adnexal structures of the dermis, can occasionally be found in the glans and prepuce, and these have been mistakenly referred to as Tyson's “glands” (12); however, they do not contribute to foreskin secretions (11, 12). Thus, any moisture found on the foreskin and glans is likely derived from serum transudate originating from the rich vascular beds in the dermis of the tissues.

The shaft, foreskin, glans, coronal sulcus, and urethral meatus are the anatomic regions of the penis that contain sufficient CD4^+^ cells to be considered potential sites of HIV transmission (reviewed in reference 10). HIV readily infects T cells and dendritic cells (DCs) in the human foreskin and glans in explant cultures (13–15), but later events in virus dissemination remain unclear. To better understand the biology of penile HIV transmission, we developed an animal model by using simian immunodeficiency virus (SIV) to inoculate the penile shaft, foreskin, coronal sulcus glans, and urethral meatus of mature male rhesus macaques (16, 17). This model recapitulates the key virologic and epidemiologic features of HIV transmission in men, including the findings that transmission is most efficient when the inoculum is placed into the subpreputial cavity rather than only onto the glans and shaft of the penis and a single viral variant is found in plasma immediately after transmission (16, 17). Importantly, the results of the STEP trial were recapitulated with this model (17). Thus, the biology of this animal model of SIV transmission seems to faithfully reproduce the biology of penile HIV transmission in men. To characterize the target cells and dissemination pathways involved in establishing systemic SIV infection, we necropsied male rhesus macaques at 1, 3, 7, and 14 days after penile SIV inoculation and determined the levels of unspliced SIV RNA (viral RNA [vRNA]) and spliced SIV RNA (mRNA) in tissue lysates and counted the number of vRNA-positive (vRNA^+^) cells in tissue sections. We found that T cells, macrophages, and dendritic cells in the glans, coronal sulcus, foreskin, and urethral transformation zone are the primary targets of infection and that SIV rapidly reaches the regional lymph nodes (LNs). The infection is initially amplified in the genital lymph nodes, and then the virus disseminates to the blood, systemic lymph nodes, and mucosal lymphoid tissues. Thus, HIV vaccines that elicit antiviral immunity and avoid inflammation and T cell activation at mucosal surfaces and mucosal lymph nodes would likely be best able to block virus dissemination after exposure.

## MATERIALS AND METHODS

### Animals.

The mature (age, >5 years) male rhesus macaques (Macaca mulatta) used in the study were housed at the California National Primate Research Center (CNPRC) in accordance with the American Association for Accreditation of Laboratory Animal Care standards. The experiments were approved by the Institutional Animal Care and Use Committee of the University of California, Davis, Davis, CA. All animals were negative for antibodies to HIV type 2 (HIV-2), SIV, type D retrovirus, and simian T cell lymphotropic virus type 1 at the time that the study was initiated.

### Penile SIV inoculation.

The pathogenic SIVmac251 stock used in this study was produced in rhesus peripheral blood mononuclear cells as previously described and contained approximately 10^5^ 50% tissue culture infective doses/ml of infectious virus and approximately 10^9^ vRNA copies/ml (16). The 2,2′-dithiodipyridine (aldrithiol-2 [AT-2])-inactivated SIVmac239 strain used in this study (SIVmac239*/SuPT1-CCR5 CL.30, lot P4266; Leidos Biomedical Research, Frederick, MD) was prepared as previously described (18, 19) and contained approximately 454 μg of total capsid, 3.5 mg of total protein, and approximately10^12^ vRNA copies/ml. The nucleocapsid (NC) protein of retroviruses contains a zinc finger sequence (Cys-X2-Cys-X4-His-X4-Cys) that is essential for the reverse transcription of genomic RNA (gRNA) to double-stranded DNA early in the virus infection cycle and for recognition and packaging of the gRNA during virion assembly. Inactivation of the zinc finger domain of NC by the compound AT-2 eliminates HIV-1 and SIV infectivity, while viral and host cell-derived proteins on virion surfaces retain their conformational and functional integrity (18, 19). Whole SIV virions chemically inactivated with AT-2 bind to CD4^+^ target cells and mediate CD4-dependent fusion comparably to native virions. However, the viral life cycle of AT-2-treated virions is arrested before initiation of reverse transcription (20), and no mRNA is produced. The virus inoculation procedure consisted of penile immersion in 2 ml of undiluted SIVmac251 stock or AT-2-inactivated SIVmac239 stock, exposing the foreskin, glans, urethral meatus, and penile shaft to the virus for 1 h. This procedure was repeated twice in 1 day with a 4-h interval between inoculations.

### Tissue collection and sample preparation.

Genital tract tissues (foreskin, glans, penis shaft, and urethral meatus) and genital lymph nodes (inguinal, obturator, and iliac lymph nodes), gut tissues (jejunum, ileum, colon, and mesenteric lymph nodes), distal lymphoid tissues (axillary lymph nodes and spleen), and blood were collected at the time of necropsy and analyzed for vRNA levels. Tissues were stored in RNAlater (Ambion, Austin, TX) and kept at −20°C until preparation of RNA. Tissue samples were homogenized with a Mini-beadbeater (Biospec Products, Bartlesville, OK) according to the manufacturer's protocol. Total RNA was isolated using the TRIzol reagent (Invitrogen, Carlsbad, CA) following the manufacturer's suggested protocol. RNA samples were treated with DNase (DNA-free; Ambion) for 1 h at 37°C. cDNA was prepared from 1 μg of tissue RNA using random primers (Invitrogen) and SuperScript III reverse transcriptase (RT; Invitrogen).

### Virologic analysis.

RT-PCR was used to detect and quantify unspliced SIV *gag* RNA and spliced SIV *rev*, *tat*, *nef*, and *vif* mRNA levels in tissue samples. The primer pairs and probes used for the assays are shown in Table 1. The SIV *gag* RNA and spliced SIV *rev*, *tat*, *nef*, and *vif* mRNA RT-PCR assays were run in separate wells, and the spliced mRNA assay was multiplexed to detect the *rev*, *tat*, *nef*, and *vif* targets simultaneously. Thus, the spliced mRNA assay does not distinguish the levels of individual spliced mRNA variants. All the reverse primers for spliced viral mRNA span at least one splice acceptor and one splice donor site (Table 1). Thus, the *vif* PCR product crosses the SD1/SA1 splice junction (21, 22), the *tat* PCR product spans the SD1/SA4^SD3/SA8 splice junctions (21, 22), the *rev* PCR products span the SD1/SA6^SD3/SA7, SD1/SA6^SD3/SA8, SD1/SA5^SD3/SA7, and SD1/SA5^SD3/SA8 splice junctions (21, 22), and the *nef* PCR products span the SD1/SA7 and SD1/SA8 splice junctions (21, 22).

**TABLE 1:** RT-PCR assays to detect unspliced and spliced SIV RNA

Target RNA	Sequence (5′–3′)			Amplicon size (bp)	Splice junction^*a*^
	Forward primer	Reverse primer	Probe		
SIV *gag* RNA	GGGAGATGGGCGTGAGAAA	CGTTGGGTCGTAGCCTAATTTT	TCATCTGCTTTCTTCCCTGACAAGACGGA	78	None
SIV *tat* mRNA	GGTCGGTACCAGACGGCGT	GTTGGCAGTGCCGGGTCCTGTTG	CTGCAACCGGAGGCCTCTTCCTCTCCC	386	SD1/SA4^SD3/SA8
SIV *rev* mRNA	GGTCGGTACCAGACGGCGT	GTTGGCAGTGCCGGGTCCTGTTG	CTGCAACCGGAGGCCTCTTCCTCTCCC	171	SD1/SA6^SD3/SA7
SIV *rev* mRNA	GGTCGGTACCAGACGGCGT	GTTGGCAGTGCCGGGTCCTGTTG	CTGCAACCGGAGGCCTCTTCCTCTCCC	174	SD1/SA6^SD3/SA8
SIV *rev* mRNA	GGTCGGTACCAGACGGCGT	GTTGGCAGTGCCGGGTCCTGTTG	CTGCAACCGGAGGCCTCTTCCTCTCCC	293	SD1/SA5^SD3/SA7
SIV *rev* mRNA	GGTCGGTACCAGACGGCGT	GTTGGCAGTGCCGGGTCCTGTTG	CTGCAACCGGAGGCCTCTTCCTCTCCC	296	SD1/SA5^SD3/SA8
SIV *nef* mRNA	GGTCGGTACCAGACGGCGT	GTTGGCAGTGCCGGGTCCTGTTG	CTGCAACCGGAGGCCTCTTCCTCTCCC	85	SD1/SA7
SIV *nef* mRNA	GGTCGGTACCAGACGGCGT	GTTGGCAGTGCCGGGTCCTGTTG	CTGCAACCGGAGGCCTCTTCCTCTCCC	88	SD1/SA8
SIV *vif* mRNA	GGTCGGTACCAGACGGCGT	CTTTAAGATGACTGCTCCTTCCC	CTGCAACCGGAGGCCTCTTCCTCTCCC	126	SD1/SA1

a mRNA splice junctions (21, 22) spanned by an amplicon.

Unless otherwise noted, all RNA samples isolated from tissues were tested in 4 replicate PCRs carried out in 96-well optical plates (Applied Biosystems, Foster City, CA). For the unspliced SIV *gag* RNA assay, each 25-μl PCR mixture contained 5 μl cDNA (equivalent to 83.3 ng total tissue RNA), 12.5 μl 2× Universal PCR master mix (Applied Biosystems), and 0.3 μM forward primer, 0.3 μM reverse primer, and 0.2 μM probe. For the spliced SIV *rev*, *tat*, *nef*, and *vif* mRNA assay, each 25-μl PCR mixture contained 5 μl cDNA, 12.5 μl 2× Universal PCR master mix, and 0.32 μM forward primer, 0.16 μM each reverse primer, and 0.2 μM probe. All PCRs were performed using an ABI 7900 robotic thermal cycler, and each run consisted of 2 min at 50°C and then 10 min at 95°C followed by 45 cycles of 15 s at 95°C and 1 min at 60°C. All PCR mixtures included primers and probes for GAPDH (glyceraldehyde-3-phosphate dehydrogenase) to detect problems with PCR or RNA isolation, and all plates contained several wells that held only 25 μl nuclease-free water to control for contamination.

The results were analyzed with SDS 7900 system software (version 2.3; Applied Biosystems). The results for the unspliced SIV *gag* RNA assay are reported as the log_10_ number of vRNA copies per microgram of tissue RNA. Because multiple spliced products of several sizes were amplified in each reaction, the results of the spliced SIV *rev*, *tat*, *nef*, and *vif* mRNA assay are reported as the threshold cycle (*C_T_*) rather than copy numbers. The *C_T_* is the PCR amplification cycle where the specific PCR signal exceeds the background signal. The lower that the *C_T_* value for a sample is, the higher that the concentration of the target sequence in the sample is. To evaluate the specificity and determine the background signals of these PCR assays, RNA isolated from 11 individual tissue samples from 6 rhesus macaques that had never been exposed to SIV was analyzed. There was no amplification of SIV *gag* from the RNA isolated from any of these tissue samples. To be conservative, 50 SIV *gag* copies/μg of tissue RNA was used as the cutoff for determining whether a tissue sample from an SIV-inoculated monkey was positive. However, the sensitivity of both SIV RNA assays is 10 copies for any single target, as determined by serial dilution of plasmids with inserts that contain the target sequences.

### Detection of SIV RNA^+^ cells in tissue sections by ISH.

RNA *in situ* hybridization (ISH) was performed to detect SIV RNA-positive cells using an RNAscope (version 2.0) high-definition (brown) kit (Advanced Cell Diagnostics, Hayward, CA) following the manufacturer's instructions (23), with modifications. Briefly, 5-μm sections from paraffin-embedded tissues fixed in 4% paraformaldehyde (Electron Microscopy Sciences, Hatfield, PA) were baked for 1 h at 60°C, deparaffinized, and dehydrated, and the slides were incubated with the pretreatment solutions provided by the manufacturer (Advanced Cell Diagnostics, Hayward, CA). The sections were incubated at 40°C for 2 h with double-Z oligonucleotide probes (Advanced Cell Diagnostics, Hayward, CA) that were designed to hybridize to the *nef*, *gag*, and *pol* RNA sequences of SIVmac239. After signal preamplification, amplification, and washes, chromogenic detection of the hybridized probe was performed using 3,3′-diaminobenzidine (DAB). The slides were counterstained with 50% Gill's hematoxylin for 2 min. Controls for the detection of a nonspecific signal included replicate sections treated with RNase A (10 μg/ml in 2× saline-sodium citrate buffer; Thermo Fisher Scientific, Waltham, MA) for 1 h at 37°C before hybridization, sense double-Z oligonucleotide probe robes, and slides of matched tissues from SIV-negative animals. All the control slides gave appropriate results.

To enumerate the SIV RNA^+^ cells in a tissue, five or more stained slides from a single paraffin block were examined on a Zeiss Imager Z1 microscope (Carl Zeiss Inc., Thornwood, NY). For each tissue, the total number of SIV-positive cells found and the total area of tissue examined (approximately 6 cm^2^) were recorded. A cell was considered SIV RNA^+^ if unequivocal brown DAB precipitates were found in the nucleus and/or cytoplasm. The data are reported as the number of SIV RNA^+^ cells per 10 cm^2^ of tissue.

### Immunophenotype of SIV RNA^+^ cells in tissue sections.

To determine the types of cells that were SIV RNA^+^, we used a method that combines ISH and immunofluorescent antibody staining (IFA) as previously described (24). The ISH protocol described above was used, except that hematoxylin counterstaining was omitted and the slides were washed with Tris buffer (pH 7.4). Then, the slides were incubated with 10% normal goat serum in 5% bovine serum albumin (BSA) to block background staining. The slides were incubated with monoclonal antibodies reactive to CD3 (made in rat; University of California, Davis, Davis, CA), fascin/p55 (made in rabbit; Abcam, Cambridge, MA), and anti-CD68 (made in mouse; Thermo Fisher Scientific, Waltham, MA). Goat anti-rabbit IgG conjugated with Alexa Fluor 488, goat anti-rat IgG conjugated with Alexa Fluor 568, and goat anti-mouse IgG conjugated with Alexa Fluor 647 (Molecular Probes, Grand Island, NY) were used to detect p55-positive (p55^+^) dendritic cells, CD3^+^ T lymphocytes, and CD68^+^ macrophages, respectively. Coverslips were placed on all slides using the ProLong Gold reagent with 4′,6-diamidino-2-phenylindole (DAPI) as a nuclear stain (Molecular Probes, Grand Island, NY).

The slides were viewed and images were captured with bright light and epifluorescent illumination using a Zeiss Imager Z1 microscope (Carl Zeiss Inc., Thornwood, NY) with appropriate filters. To capture images, SIV RNA^+^ cells were found with bright field illumination to detect DAB precipitates. Then, images of the three Alexa Fluor dyes and DAPI (blue) were taken with epifluorescent illumination and saved as separate image layers. Finally, images of the DAB precipitates from the ISH reaction in the same field were captured under a bright light. Adobe Photoshop CS5 software and OpenLab software (Inprovision Inc., Waltham, MA) were used to process the images. The CD68 signal of the Alex Fluor 647 channel was pseudocolored into yellow, the DAB staining was pseudocolored into cyan, and the background of the bright-field image was made black for overlay with the other layers of the fluorescence images. The five layers of images of each section (DAB, three Alexa Fluor dyes, and DAPI) captured were merged into red, green, and blue planes. The immunophenotype of SIV RNA^+^ cells was judged under a microscope where the planes of focus could be adjusted and by examining the layers of each captured image where the colocalization of various signals could be more precisely judged.

### Data analysis.

GraphPad Prism (version 5) software for Apple OSX11.3 (GraphPad Software, San Diego, CA, USA) and Macintosh computers (Apple Inc., Cupertino, CA) were used for statistical analysis and graphing of the data.

## RESULTS

### Timing of systemic infection after penile SIVmac251 inoculation.

**FIG 1:**
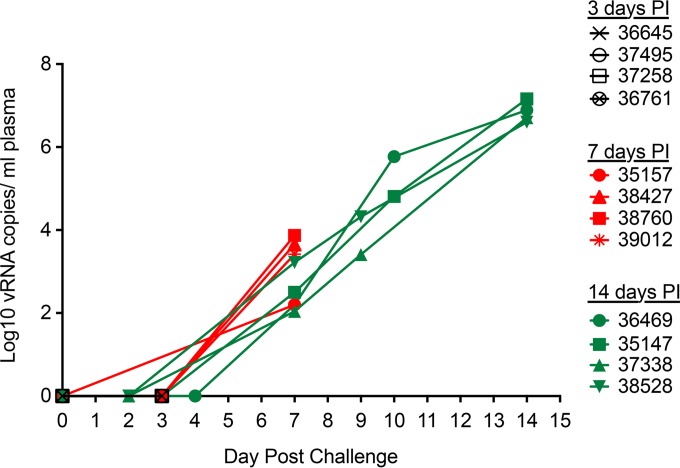
SIV RNA levels in plasma of rhesus macaques after penile SIVmac251 inoculation. Total vRNA levels in plasma were determined by RT-PCR. The last blood sample was collected at necropsy. The animal number associated with each symbol is indicated.

At 1, 3, 7, and 14 days postinoculation (p.i.), four mature male rhesus macaques were necropsied on each day and tissues were collected to determine the anatomic sites of SIV infection and replication. Plasma was also collected, and plasma vRNA was used as a marker of systemic dissemination. SIV RNA was undetectable in the plasma samples collected at 1 and 3 days p.i. (Fig. 1). At 7 days p.i., plasma vRNA levels were low (10^2^ to 10^4^ copies/ml), but by 14 days p.i. the plasma vRNA levels were very high (>10^6^ to 10^7^ copies/ml) (Fig. 1). Thus, SIV had systemically disseminated by 7 days p.i., with considerable amplification of the infection occurring from day 7 to day 14 p.i. This result is consistent with the results of previous penile SIV inoculation experiments (16); however, it is worth noting that peak plasma vRNA levels occurred at day 17 p.i. in many animals in these longer-term studies.

### Anatomic distribution of vRNA^+^ tissues and vRNA^+^ cells at 1 and 3 days p.i.

To establish the distribution of SIV following penile inoculation, a TaqMan PCR assay targeting unspliced SIV *gag* was used to detect vRNA in tissue. Because this assay cannot distinguish genomic RNA (gRNA) in virions or infected cells from SIV *gag* mRNA, we also used an RT-PCR assay to determine the levels of spliced SIV mRNAs (Table 1). Spliced SIV mRNAs are produced only in infected cells. Thus, we assumed that the unspliced vRNA indicated the presence of SIV, while spliced vRNA was a marker of active virus replication. ISH was used to determine the distribution of vRNA^+^ cells in tissues. Finally, to determine the extent to which virions in the inoculum contribute to the unspliced vRNA detected by the ISH and PCR assays, tissues from animals necropsied at 1 and 3 days after penile inoculation with AT-2-inactivated SIV were analyzed in parallel. AT-2-treated viruses fuse with the same CD4^+^ target cells (DCs, T cells) as native SIV (25), but they cannot replicate to produce spliced viral mRNA.

**FIG 2:**
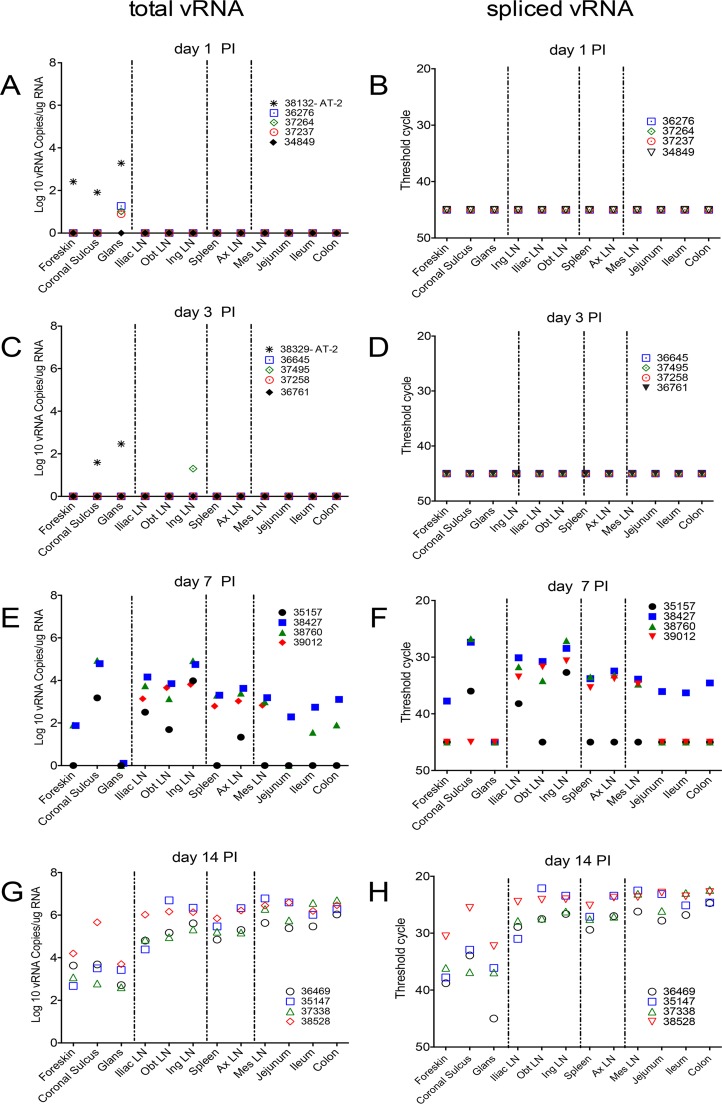
Unspliced and spliced vRNA levels in tissues of rhesus macaques after penile SIV inoculation. (A, C, E, and G) The level of unspliced vRNA, representing gRNA in virions and infected cells and the bulk of the SIV mRNA in infected cells, is shown; (B, D, F, and H) spliced vRNA is only a fraction of the SIV mRNA in infected cells, but it is an unambiguous marker of transcription from integrated SIV DNA provirus. Tissues were collected on the indicated days postinoculation. The animal number associated with each symbol is indicated. The dashed vertical lines divide the tissues into 4 anatomic regions. From left to right, the regions are (i) regions of the penis, (ii) genital lymph nodes, (iii) systemic lymphoid tissues, and (iv) gastrointestinal tract and draining mesenteric LN. Obt LN, obturator LN; Ing LN, inguinal LN; Ax LN, axillary LN; Mes, mesenteric LN.

At day 1 p.i., unspliced vRNA was detected at low to moderate levels in the coronal sulcus, foreskin, and glans of an animal inoculated with AT-2-treated SIV (animal 38132). In addition, unspliced vRNA was detected at very low levels in the glans of 3 of the animals inoculated with SIVmac251 (Fig. 2A). However, spliced vRNA was not detected in any tissue from the 4 animals necropsied 1 day after SIVmac251 inoculation (Fig. 2B). In the animals necropsied at day 3 p.i., a very low level of unspliced vRNA was detected in the inguinal lymph node (LN) of 1 of the animals inoculated with SIVmac251. In addition, vRNA was detected in the coronal sulcus and glans of an animal inoculated with AT-2-treated SIV (animal 38329) (Fig. 2C). However, spliced vRNA was not detected in any tissue from the 4 animals necropsied 3 days after SIVmac251 inoculation (Fig. 2D). These results suggest that at 24 h after penile inoculation, virions in the inoculum persist in genital surfaces but little virus replication has occurred. SIV RNA was similarly distributed at 3 days p.i., with some evidence of vRNA dissemination to the draining lymph nodes being found in 1 animal, but viral replication was insufficient for spliced vRNA to be detectable with our assays.

**FIG 3:**
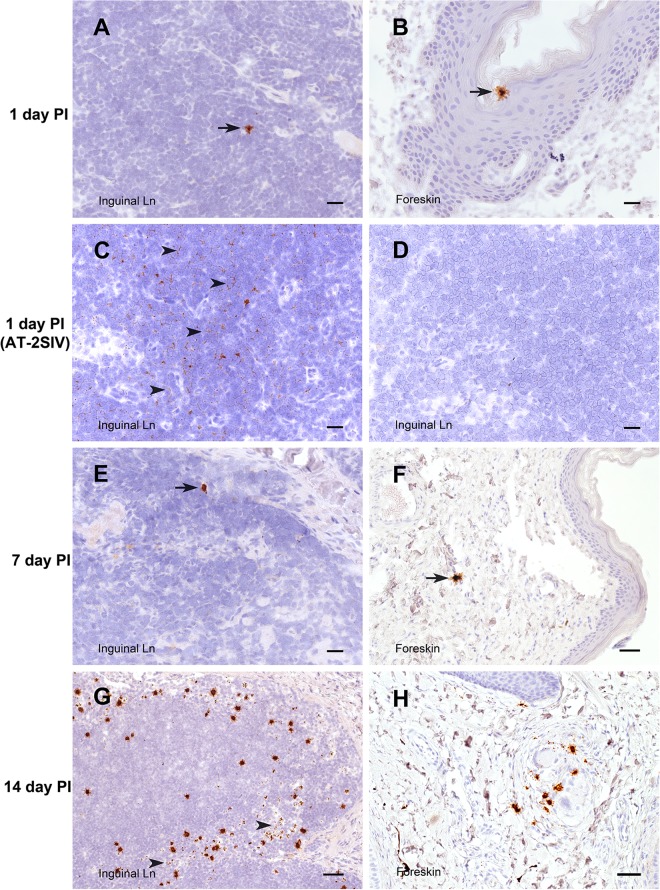
SIV RNA^+^ cells in inguinal LN and foreskin. Cells containing vRNA (brown) were detected in tissue sections using *in situ* hybridization with antisense SIV-specific riboprobes. The interval between the time of SIV inoculation and the time of tissue collection is indicated for each row. (A, B, and E to H) Sections of tissue from animals inoculated with SIVmac251 hybridized with antisense SIV-specific riboprobes. (C and D) Sections of tissue from an animal inoculated with AT-2-inactivated SIV and necropsied at 1 day p.i. (C) A normally processed section hybridized with antisense SIV-specific riboprobes. (D) RNase treatment prior to hybridization with antisense SIV-specific riboprobes. Arrowheads in panel C, areas in the section with abundant vRNA staining in a faint reticular pattern that is consistent with extracellular SIV virion RNA; arrowheads in panel G, 3 of the few SIV RNA^+^ cells within the T cell zone of the LN. Bars = 20 μm (A to E), 50 μm (F, H), and 100 μm (G). A DAB label and hematoxylin counterstain were used.

**FIG 4:**
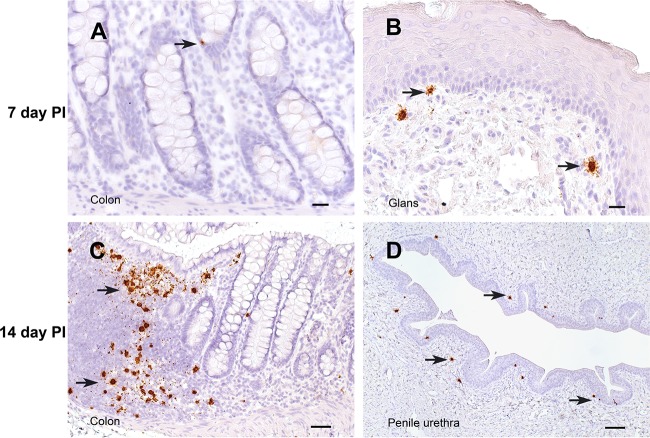
SIV RNA^+^ cells in the penis and colon. The interval between the time of SIV inoculation and the time of tissue collection is indicated for each row. All panels show sections of tissues collected from animals inoculated with SIVmac251 hybridized with antisense SIV-specific riboprobes. Arrows, representative vRNA^+^ cells in each section. Bars = 20 μm (A, B), 50 μm (C), and 100 μm (D). A DAB label and hematoxylin counterstain were used.

**FIG 5:**
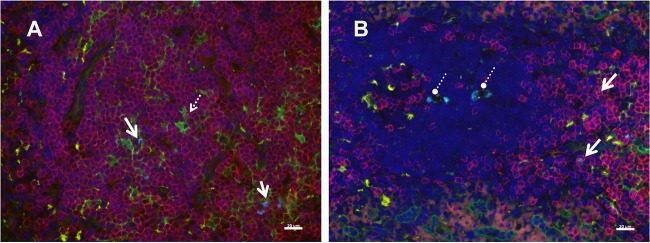
Immunophenotype of SIV RNA^+^ cells 24 h after SIVmac251 inoculation. Cells containing vRNA were detected in tissue sections using *in situ* hybridization with fluorescently tagged antisense SIV-specific riboprobes and antibodies to phenotype cells using cell markers in tissues at 24 h p.i. (A) Inguinal lymph node; (B) spleen. Solid arrows, representative SIV RNA^+^ T cells (bright blue); these are often associated with p55^+^ (fascin-positive) DCs; dashed arrow in panel A, a vRNA^+^ T cell abutting a p55^+^ DC; circles with dashed lines in panel B, vRNA within macrophages. The pattern of vRNA in the macrophage cytoplasm (several discrete vRNA^+^ foci) is in a pattern consistent with the phagocytosis of vRNA^+^ T cells. Red, CD3^+^ T cells; green, p55^+^ endothelial cells and bone marrow-derived DCs; yellow, CD68^+^ macrophages; bright blue, SIV RNA; dark blue, DAPI staining of nuclear DNA. Bars = 20 μm.

**FIG 6:**
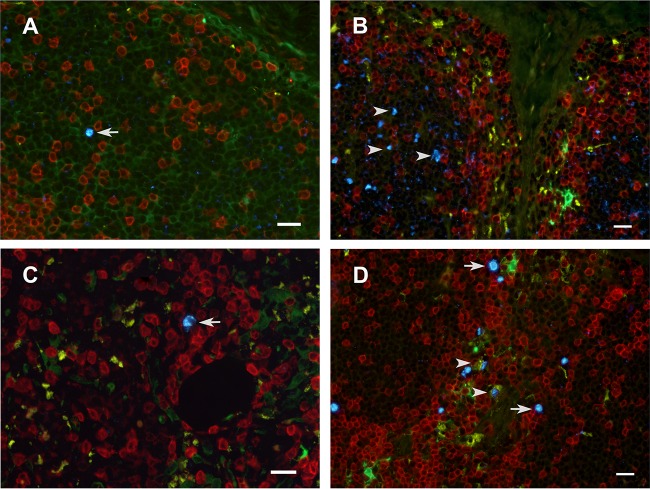
Immunophenotype of SIV RNA^+^ cells in the inguinal LN. Cells containing vRNA were detected in tissue sections using *in situ* hybridization with fluorescently tagged antisense SIV-specific riboprobes and antibodies to phenotype cells using cell markers. The images are from 1 day p.i. (A and B), 7 days p.i. (C), and 14 days p.i. (D) (A, C, D) Tissues collected from animals inoculated with SIVmac251; (B) tissue collected from an animal inoculated with AT-2-inactivated SIV and necropsied at 1 day p.i. Arrows, representative SIV RNA^+^ cells (bright blue) located mostly in the T cell-rich paracortex (blue); arrowheads in panel B, extracellular vRNA within a B cell follicle; arrowheads in panel D, vRNA within macrophages. The vRNA signal is separated by a clear space from the surrounding macrophage cytoplasm, as if it were in a phagosome. Red, CD3^+^ T cells; green, p55^+^ endothelial cells and bone marrow-derived DCs; yellow, CD68^+^ macrophages; bright blue, SIV RNA; dark blue, DAPI staining of nuclear DNA. Bars = 20 μm.

**FIG 7:**
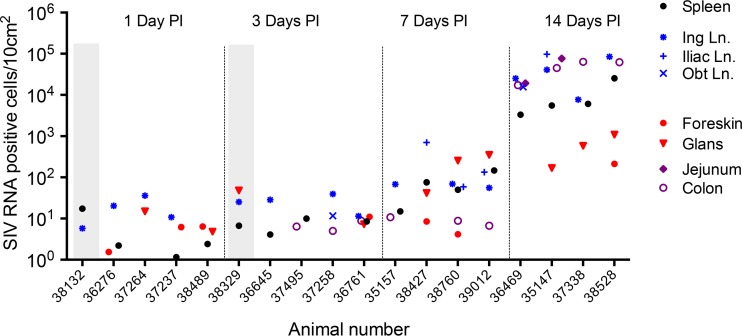
Density of SIV RNA^+^ cells in tissues. Cells containing vRNA were detected in tissue sections using *in situ* hybridization. The number of cells in >5 tissue sections of known surface area were counted, and the results are expressed as the number of SIV RNA^+^ cells/10 cm^2^. Each symbol corresponds to the indicated tissue. Red, genital tract; blue, genital LNs; black, systemic lymphoid tissue (spleen); purple, GI tract. Results from the animals inoculated with AT-2-inactivated SIV are within the vertical gray columns at 1 and 3 days p.i. Note that not every tissue was assessed in every animal, but all available results are shown.

Very sensitive *in situ* hybridization targeting unspliced vRNA confirmed the results of the RT-PCR assay (Fig. 3 to 7). At 1 and 3 days after inoculation with SIVmac251, 7 of 8 animals had rare but detectable vRNA^+^ cells in at least 1 region of the penis and vRNA^+^ cells were found in the genital lymph node of 6 of 8 animals (Fig. 3A and B and 7). While most of the vRNA^+^ cells in the penis were found in the lamina propria underlying a squamous epithelium, SIV RNA^+^ cells were also occasionally found within the epithelium of the foreskin and glans (Fig. 3B). The vRNA^+^ cells in the genital lymph nodes were found in the subcapsular sinuses and T cell zones (Fig. 3A). Notably, vRNA^+^ cells were found in the spleen from 6 of the 8 animals necropsied at days 1 and 3 p.i. and in the colon of 3 of 4 animals necropsied at day 3 p.i. (Fig. 7). At day 1 p.i., about half of the vRNA^+^ cells in tissues were CD3^+^ T cells, and the rest of the vRNA^+^ cells were CD3^−^ DCs and macrophages (Table 2; Fig. 5 and 6A). At day 3 p.i., about a third of the vRNA^+^ cells in tissues were CD3^+^ T cells, a third were macrophages, and the rest of the cells were evenly divided between DCs and unidentified/unlabeled cells (Table 2; Fig. 6B).

**TABLE 2:** Immunophenotype of SIV RNA^+^ cells in tissues

Day	Total area examined (cm^2^)^*a*^	No. (%) of cells				
		CD3^+^ (T cells)^*b*^	p55^+^ (DCs)^*c*^	CD68^+^ (macrophages)^*d*^	Other^*e*^	Total SIV RNA^+^ cells^*f*^
1	2.36	8 (50)	6 (37.5)	1 (6.25)	1 (6.25)	16
3	2.65	11 (36.7)	4 (13.3)	12 (40)	3 (10)	30
7	1.52	14 (63.6)	2 (9.1)	6 (27.3)	0	22

a The total surface area of all the tissue sections examined by quantitative cell staining at each time point. Multiple sections of spleen, inguinal LN, obturator LN, iliac LN, glans, foreskin, and coronal sulcus tissues from all SIVmac251-inoculated animals were examined, and the aggregate surface area of all the sections is reported. Note that the colon and jejunum were not included in the analysis are presented here, and this should be considered when reconciling the results reported in this table with those reported in Fig. 7.

b The number of SIV RNA^+^/CD3^+^ T cells found in all the tissues that were examined. The percentage of the total SIV RNA^+^ cells that were T cells is given in parentheses.

c The number of SIV RNA^+^/p55^+^ DCs found in all the tissues that were examined. The percentage of the total SIV RNA^+^ cells that were DCs is given in parentheses.

d The number of SIV RNA^+^/CD68^+^ macrophages found in all the tissues that were examined. The percentage of the total SIV RNA^+^ cells that were macrophages is given in parentheses.

e The number of SIV RNA^+^/CD3^−^/p55^−^/CD68^−^ cells found in all the tissues that were examined. The percentage of the total SIV RNA^+^ cells that were CD3^−^/P55^−^/CD68^−^ cells is given in parentheses.

f The number of SIV RNA^+^/CD3^−^/p55^−^/CD68^−^ cells found in all the tissues that were examined. The percentage of the total SIV RNA^+^ cells that were CD3^−^/p55^−^/CD68^−^ cells is given in parentheses.

Of note, vRNA was also found in the tissues of animals inoculated with AT-2-inactivated SIV (Fig. 3C and D); however, the staining pattern was very distinct. In tissues from SIVmac251-inoculated animals, the staining was mostly of discrete individual cells, with the vRNA signal often being concentrated in the nucleus of vRNA^+^ cells (Fig. 3A and B). In the animals inoculated with AT-2-inactivated SIV, the vRNA signal was diffuse, appeared to be extracellular, and formed a reticular pattern that was not readily associated with a cell body (Fig. 3C and 6B). Pretreatment of the sections with RNase eliminated the staining (Fig. 3D), confirming that the ISH signal was due to the presence of SIV RNA in the sections. Thus, both extracellular SIV RNA in virions and intracellular SIV RNA can be detected by the ISH assay.

### Anatomic distribution of vRNA^+^ tissues and vRNA^+^ cells at 7 days p.i.

At day 7 p.i., the point at which vRNA is first detectable in plasma after penile SIVmac251 inoculation, unspliced vRNA and spliced vRNA were present at low to moderate levels in genital tissues from 3 of 4 of the animals (Fig. 2E and F). Further, unspliced vRNA and spliced vRNA were found at moderate levels in the LNs that drain the genital tissues (iliac LN, obturator LN, inguinal LN) and at lower levels in systemic lymphoid tissues (spleen, axillary LN) of all 4 animals (Fig. 2E and F). However, unspliced vRNA was present in the gastrointestinal (GI) tract of only 2 of 4 animals, and spliced vRNA was present in the GI tract of only 1 animal. In contrast, unspliced vRNA and spliced vRNA were found at moderate levels in the mesenteric LN of 3 of the 4 animals (Fig. 2E and F), suggesting that virus replication is more rapidly established in GI tract lymph nodes than in the intestine proper. At 7 days after SIVmac251 inoculation, vRNA^+^ cells were found in the genital tissues, spleen, and lymph nodes (Fig. 3E and F, 4B, and 7) and, less frequently, in the colon (Fig. 4A and 7). At day 7 p.i., most (64%) of the vRNA^+^ cells in these tissues were CD3^+^ T cells, but a quarter of the vRNA^+^ cells were CD68^+^ macrophages and 10% were p55^+^ DCs (Table 2; Fig. 6B).

### Anatomic distribution of vRNA^+^ tissues and vRNA^+^ cells at 14 days p.i.

At day 14 p.i., unspliced vRNA and spliced vRNA were present at low to moderate levels in genital tissues and moderate to high levels in the genital LNs and systemic lymphoid tissues of all 4 animals (Fig. 2G and H and 7). However, at day 14 p.i. the highest levels of unspliced vRNA and spliced vRNA among the tissues examined in this study were found in the GI tract and mesenteric LN of the animals (Fig. 2G and H).

At 14 days p.i., vRNA^+^ cells were readily detectable in the genital tract, lymph nodes, spleen, and GI tract of most animals inoculated with SIVmac251 (Fig. 3G and H, 4C and D, and 7). At day 14 p.i., most of the vRNA^+^ cells were found in T cell zones of organized lymphoid tissues (lymph nodes and mucosa-associated lymphoid tissues), and fewer vRNA^+^ cells were associated with the diffuse lymphoid tissues of mucosal surfaces or B cell follicles of lymphoid tissues. At day 14 p.i., most of the vRNA^+^ cells in these tissues were CD3^+^ (Fig. 6D), but a minor population of CD3^−^ vRNA^+^ cells was also consistently found in lymphoid tissues, including what appeared to be macrophages that had phagocytized SIV RNA^+^ T cells (Fig. 6D) (26).

## DISCUSSION

The goal of this study was to characterize the biology of penile SIV transmission by defining the target cells involved in transmission across the epithelial barriers, the tissues where the infection is amplified, and the anatomic pathways of virus dissemination from the epithelial surfaces of the penis to the bloodstream and systemic lymphoid tissues. Using sensitive RT-PCR and ISH assays, we found that in the first 3 days after penile SIV inoculation, vRNA and vRNA^+^ cells were very difficult to detect in any tissues, and when they were detected, they were present only at low levels. While unspliced RNA was detectable in penile tissues and draining lymph nodes at days 1 and 3 p.i., it was detected at very low levels, and spliced SIV mRNA, indicative of active virus replication, was never detected. Of note, at days 1 and 3 p.i., the highest levels of unspliced vRNA were found in the penile tissues collected from the 2 animals inoculated with AT-2-inactivated SIV, clearly demonstrating that the unspliced vRNA detected at these early time points was not a product of *de novo* virus replication. In contrast, after rectal SIVmac251 inoculation, spliced vRNA was detected in the mucosa but not the draining lymph nodes of 3 of 9 macaques necropsied at between 4 h and 3 days p.i. (27). The relative delay in the detection of spliced SIV mRNA in tissues after penile inoculation is consistent with observations that penile SIV transmission is less efficient than vaginal SIV transmission, and in the animals that become infected after penile inoculation, the plasma vRNA level peaks at a time days later than the time to peak plasma vRNA levels after vaginal and rectal SIVmac251 inoculation (16, 28, 29).

ISH-based detection of SIV RNA^+^ cells in tissue sections largely confirmed the results of the PCR analysis, as the highest concentration of vRNA^+^ cells at days 1 and 3 p.i. was in the penile tissues and inguinal lymph node. However, vRNA^+^ cells were also detected by ISH in the spleens of 6 of 8 animals and the colons of 3 of 4 animals necropsied within 3 days of SIVmac251 inoculation. Further, vRNA^+^ cells were found in the spleens of both rhesus macaques inoculated with AT-2-inactivated SIV, confirming that *de novo* virus replication is not required for SIV RNA^+^ cells to reach the spleen in the first few days after penile inoculation. In fact, the vRNA^+^ cells in the spleen at days 1 and 3 p.i. may be migratory phagocytes that accumulated virions present in the inoculum at penile mucosal surfaces and then traveled to the spleen. The route used by the SIV RNA^+^ cells to reach the spleen within 24 h p.i. is unclear, and such rapid dissemination into the systemic lymphoid tissues could be consistent with direct inoculation of virus into blood vessels and trapping of free virions circulating in blood by splenic reticuloendothelial cells. However, plasma vRNA was undetectable in the animals necropsied at day 1 (data not shown) or day 3 p.i. In fact, cells can move up the lymphoid chain and into the bloodstream quickly; labeled lymphocytes infused into the thoracic duct of rats were found to reach the spleen in less than 100 min (30). Although this journey would require more time in larger animals, it seems likely that vRNA^+^ cells from the genital tract or draining lymph nodes of a rhesus macaque could ascend the lymphoid chain to the thoracic duct and recirculate to the spleen in 24 h.

By day 7 p.i., SIV RNA had disseminated to every anatomic site examined in 2 of 4 animals, and in all 4 animals, the highest levels of spliced and unspliced vRNA were found in the genital lymph nodes. The high levels of spliced vRNA indicate that the genital lymph nodes and especially the most proximal inguinal lymph node are the primary site of virus replication in the first week after penile SIV transmission. This is the site where the SIV infection is amplified and becomes established in the host. At day 14 p.i., spliced and unspliced SIV RNA levels were high in every anatomic site in all animals examined, but they were the highest in the GI tract and genital lymph nodes. Thus, at day 14 p.i., SIV replication was concentrated in these tissues. The penile tissues had the lowest levels of vRNA among the tissues examined at day 14 p.i.

Compared to the findings obtained after vaginal and rectal SIV inoculation (27, 31–33), the finding that very little vRNA and few vRNA^+^ cells are detectable in tissues in the first days p.i. is consistent with our observation that penile SIV transmission is inefficient compared to vaginal SIV transmission (16). In fact, only a single SIV *env* variant was found to be transmitted by penile SIVmac251 inoculation (16, 29), while multiple variants were found to be responsible for founding the systemic infection after vaginal and rectal SIV inoculation (34). Thus, even with the high-titer virus stocks used for the experimental inoculations, very few virions in the inoculum penetrate the penile epithelium to replicate productively in target cells. Even after the virions have traversed the epithelial barrier, the dermis of the normal penis (in the case of no inflammation) has relatively few CD4^+^ cells (10, 35) that can support virus replication compared to the number in the rectum or vagina (27, 31, 36–38), and this paucity of target cells likely explains the inefficiency of penile SIV transmission. It is worth noting that in both men and male rhesus macaques, the CD4^+^ T cells that are in the foreskin have an effector memory phenotype and express higher levels of activation markers than blood T cells (39, 40). Although the presence of this highly susceptible cell population may explain why the foreskin is important in HIV transmission, we found low levels of vRNA and a few vRNA^+^ cells in the glans, foreskin, coronal sulcus, and shaft of the penis at 24 h p.i. HIV virions also penetrate the foreskin and glans epithelia of experimentally inoculated rhesus macaques (41). Thus, we found no evidence that one area of the penis is particularly vulnerable to transmission, suggesting that the virus can be transmitted across the epithelial surfaces at all these sites. This conclusion is consistent with the observed 50% reduction in HIV transmission that occurs after circumcision (which removes about 50% of the surface area of the penis) (4–8). While many anatomic sites seem to harbor susceptible target cells, the totality of the experimental results reported to date suggest that infection of a few CD4^+^ target cells, perhaps in a single anatomic site, establishes the clonal founding virus population (16) and the systemic infection after penile SIV inoculation.

The discordance between the results of the PCR and ISH assays with spleen and colon samples collected at days 1 and 3 p.i. cannot be explained by a technical error, as the control experiments for both assays worked as expected. Perhaps the few RNA^+^ cells in tissues at these early times were unevenly distributed in the small tissue aliquots collected for PCR and ISH assays. In fact, assuming that 10 pg RNA is present per cell (42), the 332 ng of cDNA (reverse-transcribed RNA) used for the PCR assays (83 ng cDNA/reaction mixture × 4 replicates) represents only 42,000 cells. Thus, we increased the total number of PCRs to analyze 100 μg of total RNA from selected samples collected on days 1 and 3 p.i. This allowed us to analyze 10^7^ RNA cell equivalents per tissue sample, but there was no change from the results reported in Fig. 2 (data not shown). For comparison, based on the counts of DAPI-labeled nuclei, we estimate that a typical section of spleen and lymph node tissue examined in the ISH assay contained 0.2 × 10^6^ to 2 × 10^6^ cells, while a section of colon contained 10^5^ cells (data not shown). At least 5 sections from each tissue specimen collected from the animals on days 1 and 3 p.i., which is equivalent to approximately 0.1 × 10^7^ to 1 × 10^7^ cells from each tissue specimen, were analyzed by ISH. Clearly, both assays interrogate only a small fraction of the lymphocytes in a tissue specimen, but compared to the results of our standard PCR assay, the ISH assay results are based on an analysis of up to 10 times more cells for the presence vRNA. Thus, to reduce the sampling error inherent in studies of very early stages of SIV infection, methods that can reliably detect a few copies of vRNA in a very large amount (milligrams) of tissue RNA are needed.

In all tissues examined from all animals at all time points p.i., the great majority of the vRNA^+^ cells were CD3^+^ T cells (Table 2). However, at the earliest time points, about half the vRNA^+^ cells were CD3^−^, and these were CD68^+^ macrophages, p55^+^ DCs (24), or triple-negative cells that were likely T cells with SIV *nef*-driven downregulated CD3 expression (43). While vRNA^+^ cells were rare at 1 and 3 days p.i. (Table 2), the vRNA^+^ CD3^+^ T cells were often found in close association with p55^+^ DCs and the vRNA^+^ signal seemed to be shared by the adjacent cells (Fig. 5). The high number of vRNA^+^ macrophages in tissues at days 3, 7, and 14 p.i. could be due to the phagocytosis of opsonized virions and the dying SIV-infected T cells that are detected as virus replication ramps up, but the possibility of productive virus replication in these cells cannot be ruled out. In fact, T cells can be nonproductively infected by replication-defective virions and can be vRNA^+^ without producing infectious virus. Thus, ISH likely overestimates the number of productively infected T cells as well as DCs and macrophages.

In tissue culture, macaque DCs readily take up infectious and AT-2-inactivated SIV (25), and although few intact virus particles are found in immature DCs, numerous virions are found in large vesicular compartments within mature DCs (25). Thus, the vRNA^+^ p55^+^ DCs in tissue sections of the SIV-inoculated animals may not be productively infected DCs but, rather, may be DCs with virions in vacuoles. In fact, ISH can detect cells that are vRNA^+^ due to one or more of the following conditions. They have (i) intact SIV virions in cytoplasm vacuoles (25), (ii) a transcriptionally active SIV provirus that is producing gRNA and mRNA, and (iii) phagocytized virus-infected cells (26, 44). The significance of the reticular pattern of vRNA localization in the lymph nodes of animals inoculated with AT-2-inactivated SIV is unclear but could be consistent with virion capture by dendritic cells (45). The capture of virions by DCs in the acute stage of SIV infection is consistent with reports that dendritic cells residing in the lymph node medulla use a lectin receptor to capture lymph-borne influenza virus within hours of injection (46) and could be mediated by DC-specific intercellular adhesion molecule 3-grabbing nonintegrin (DC-SIGN). DC-SIGN binds HIV-1 on the surface of DCs and macrophages, but it does not allow viral infection of the cell (47).

The results of the studies described here emphasize that penile SIV transmission occurs when a relatively few cells become infected. The results suggest that many areas of the penis, including the glans, foreskin, urethral meatus, and coronal sulcus, can be sites of SIV transmission after penile inoculation. Further, genital mucosal T cells and, to a lesser extent, DCs and macrophages seem to be the targets of infection, and these infected cells migrate to regional lymph nodes, where the infection is initially amplified. After several days and several rounds of virus replication in the genital lymph nodes, the infection travels through the abdominal and thoracic lymphatic vessels to the bloodstream. Between days 7 and 14 p.i., the virus disseminates through the blood to secondary sites of replication, including the systemic and GI tract lymphoid tissues, with dramatic increases in plasma vRNA levels being detected. All the findings presented above are based on experimental inoculation of intact, noninflamed epithelial surfaces covering the penis. The details of penile SIV transmission may be different in the setting of inflammation and the presence of concurrent sexually transmitted infections. The stepwise dissemination pathway of SIV (48) from the surface of the penis, through draining lymph nodes, and to its final target organs in the gut and systemic lymphoid tissues is similar to the dissemination process that has been described after vaginal and rectal SIV inoculation (27, 31, 33, 48), though the process seems to be up to a week slower after penile inoculation. Because the first anatomic site where SIV and, by extension, HIV replicate efficiently after penile transmission is in the draining lymph nodes. HIV vaccines that elicit in these tissues antiviral cytotoxic T lymphocytes that kill infected cells before they produce progeny virions could prevent the nascent local HIV infections from progressing to systemic infections. However, these types of vaccine strategies must also avoid inducing inflammation and T cell activation at mucosal surfaces, as those processes could increase the number of infected target cells entering the draining lymph nodes and overwhelm any antiviral immunity.

## References

[1] UNAIDS. 2014 The gap report. World Health Organization and Joint United Nations Programme on HIV/AIDS, Geneva, Switzerland.

[2] Buchbinder SP, Mehrotra DV, Duerr A, Fitzgerald DW, Mogg R, Li D, Gilbert PB, Lama JR, Marmor M, Del Rio C, McElrath MJ, Casimiro DR, Gottesdiener KM, Chodakewitz JA, Corey L, Robertson MN 2008 Efficacy assessment of a cell-mediated immunity HIV-1 vaccine (the Step study): a double-blind, randomised, placebo-controlled, test-of-concept trial. Lancet 372:1881–1893. doi:10.1016/S0140-6736(08)61591-3. 19012954 PMC2721012

[3] Joint United Nations Programme on HIV/AIDS. 2007 Male circumcision: global trends and determinants of prevalence, safety and acceptability. World Health Organization and Joint United Nations Programme on HIV/AIDS, Geneva, Switzerland.

[4] Alsallaq RA, Cash B, Weiss HA, Longini IM Jr, Omer SB, Wawer MJ, Gray RH, Abu-Raddad LJ 2009 Quantitative assessment of the role of male circumcision in HIV epidemiology at the population level. Epidemics 1:139–152. doi:10.1016/j.epidem.2009.08.001. 21352761

[5] Auvert B, Taljaard D, Lagarde E, Sobngwi-Tambekou J, Sitta R, Puren A 2005 Randomized, controlled intervention trial of male circumcision for reduction of HIV infection risk: the ANRS 1265 Trial. PLoS Med 2:e298. doi:10.1371/journal.pmed.0020298. 16231970 PMC1262556

[6] Bailey RC, Moses S, Parker CB, Agot K, Maclean I, Krieger JN, Williams CF, Campbell RT, Ndinya-Achola JO 2007 Male circumcision for HIV prevention in young men in Kisumu, Kenya: a randomised controlled trial. Lancet 369:643–656. doi:10.1016/S0140-6736(07)60312-2. 17321310

[7] Gray RH, Li X, Kigozi G, Serwadda D, Nalugoda F, Watya S, Reynolds SJ, Wawer M 2007 The impact of male circumcision on HIV incidence and cost per infection prevented: a stochastic simulation model from Rakai, Uganda. AIDS 21:845–850. doi:10.1097/QAD.0b013e3280187544. 17415039

[8] Hallett TB, Singh K, Smith JA, White RG, Abu-Raddad LJ, Garnett GP 2008 Understanding the impact of male circumcision interventions on the spread of HIV in southern Africa. PLoS One 3:e2212. doi:10.1371/journal.pone.0002212. 18493593 PMC2387228

[9] Velazquez EF, Barreto JE, Cubilla AL 2012 Penis and distal urethra. *In* Mills SE (ed), Histology for pathologists, 4th ed Lippincott (Wolters Kluwer Health), Philadelphia, PA.

[10] Anderson D, Politch JA, Pudney J 2011 HIV infection and immune defense of the penis. Am J Reprod Immunol 65:220–229. doi:10.1111/j.1600-0897.2010.00941.x. 21214659 PMC3076079

[11] Parkash S, Jeyakumar S, Subramanyan K, Chaudhuri S 1973 Human subpreputial collection: its nature and formation. J Urol 110:211–212. 4722614 10.1016/s0022-5347(17)60164-2

[12] Hyman AB, Brownstein MH 1969 Tyson's “glands.” Ectopic sebaceous glands and papillomatosis penis. Arch Dermatol 99:31–36. doi:10.1001/archderm.1969.01610190037006. 5761803

[13] Fischetti L, Barry SM, Hope TJ, Shattock RJ 2009 HIV-1 infection of human penile explant tissue and protection by candidate microbicides. AIDS 23:319–328. doi:10.1097/QAD.0b013e328321b778. 19114867 PMC4349942

[14] Ganor Y, Zhou Z, Tudor D, Schmitt A, Vacher-Lavenu MC, Gibault L, Thiounn N, Tomasini J, Wolf JP, Bomsel M 2010 Within 1 h, HIV-1 uses viral synapses to enter efficiently the inner, but not outer, foreskin mucosa and engages Langerhans-T cell conjugates. Mucosal Immunol 3:506–522. doi:10.1038/mi.2010.32. 20571487

[15] Ganor Y, Zhou Z, Bodo J, Tudor D, Leibowitch J, Mathez D, Schmitt A, Vacher-Lavenu MC, Revol M, Bomsel M 2013 The adult penile urethra is a novel entry site for HIV-1 that preferentially targets resident urethral macrophages. Mucosal Immunol 6:776–786. doi:10.1038/mi.2012.116. 23187317

[16] Ma ZM, Keele BF, Qureshi H, Stone M, Desilva V, Fritts L, Lifson JD, Miller CJ 2011 SIVmac251 is inefficiently transmitted to rhesus macaques by penile inoculation with a single SIVenv variant found in ramp-up phase plasma. AIDS Res Hum Retroviruses 27:1259–1269. doi:10.1089/aid.2011.0090. 21732792 PMC3227244

[17] Qureshi H, Ma ZM, Huang Y, Hodge G, Thomas MA, DiPasquale J, DeSilva V, Fritts L, Bett AJ, Casimiro DR, Shiver JW, Robert-Guroff M, Robertson MN, McChesney MB, Gilbert PB, Miller CJ 2012 Low-dose penile SIVmac251 exposure of rhesus macaques infected with adenovirus type 5 (Ad5) and then immunized with a replication-defective Ad5-based SIV gag/pol/nef vaccine recapitulates the results of the phase IIb step trial of a similar HIV-1 vaccine. J Virol 86:2239–2250. doi:10.1128/JVI.06175-11. 22156519 PMC3302390

[18] Rossio JL, Esser MT, Suryanarayana K, Schneider DK, Bess JW Jr, Vasquez GM, Wiltrout TA, Chertova E, Grimes MK, Sattentau Q, Arthur LO, Henderson LE, Lifson JD 1998 Inactivation of human immunodeficiency virus type 1 infectivity with preservation of conformational and functional integrity of virion surface proteins. J Virol 72:7992–8001. 9733838 10.1128/jvi.72.10.7992-8001.1998 PMC110135

[19] Arthur LO, Bess JW Jr, Chertova EN, Rossio JL, Esser MT, Benveniste RE, Henderson LE, Lifson JD 1998 Chemical inactivation of retroviral infectivity by targeting nucleocapsid protein zinc fingers: a candidate SIV vaccine. AIDS Res Hum Retroviruses 14(Suppl 3):S311–S319. 9814959

[20] Morcock DR, Thomas JA, Gagliardi TD, Gorelick RJ, Roser JD, Chertova EN, Bess JW Jr, Ott DE, Sattentau QJ, Frank I, Pope M, Lifson JD, Henderson LE, Crise BJ 2005 Elimination of retroviral infectivity by N-ethylmaleimide with preservation of functional envelope glycoproteins. J Virol 79:1533–1542. doi:10.1128/JVI.79.3.1533-1542.2005. 15650179 PMC544125

[21] van der Velden GJ, Klaver B, Das AT, Berkhout B 2012 Upstream AUG codons in the simian immunodeficiency virus SIVmac239 genome regulate Rev and Env protein translation. J Virol 86:12362–12371. doi:10.1128/JVI.01532-12. 22951834 PMC3486506

[22] Park IW, Steen R, Li Y 1991 Characterization of multiple mRNA species of simian immunodeficiency virus from macaques in a CD4^+^ lymphoid cell line. J Virol 65:2987–2992. 1674547 10.1128/jvi.65.6.2987-2992.1991 PMC240945

[23] Wang F, Flanagan J, Su N, Wang LC, Bui S, Nielson A, Wu X, Vo HT, Ma XJ, Luo Y 2012 RNAscope: a novel in situ RNA analysis platform for formalin-fixed, paraffin-embedded tissues. J Mol Diagn 14:22–29. doi:10.1016/j.jmoldx.2011.08.002. 22166544 PMC3338343

[24] Ma ZM, Miller CJ 2015 Immunophenotype of simian immunodeficiency virus-infected cells in the spleen of a rhesus monkey. AIDS Res Hum Retroviruses 31:359–360. doi:10.1089/aid.2014.0343. 25760311 PMC4378854

[25] Frank I, Piatak M Jr, Stoessel H, Romani N, Bonnyay D, Lifson JD, Pope M 2002 Infectious and whole inactivated simian immunodeficiency viruses interact similarly with primate dendritic cells (DCs): differential intracellular fate of virions in mature and immature DCs. J Virol 76:2936–2951. doi:10.1128/JVI.76.6.2936-2951.2002. 11861860 PMC135959

[26] Baxter AE, Russell RA, Duncan CJ, Moore MD, Willberg CB, Pablos JL, Finzi A, Kaufmann DE, Ochsenbauer C, Kappes JC, Groot F, Sattentau QJ 2014 Macrophage infection via selective capture of HIV-1-infected CD4^+^ T cells. Cell Host Microbe 16:711–721. doi:10.1016/j.chom.2014.10.010. 25467409 PMC4271767

[27] Ribeiro Dos Santos P, Rancez M, Pretet JL, Michel-Salzat A, Messent V, Bogdanova A, Couedel-Courteille A, Souil E, Cheynier R, Butor C 2011 Rapid dissemination of SIV follows multisite entry after rectal inoculation. PLoS One 6:e19493. doi:10.1371/journal.pone.0019493. 21573012 PMC3090405

[28] Ma ZM, Abel K, Rourke T, Wang Y, Miller CJ 2004 A period of transient viremia and occult infection precedes persistent viremia and antiviral immune responses during multiple low-dose intravaginal simian immunodeficiency virus inoculations. J Virol 78:14048–14052. doi:10.1128/JVI.78.24.14048-14052.2004. 15564513 PMC533914

[29] Keele BF, Li H, Learn GH, Hraber P, Giorgi EE, Grayson T, Sun C, Chen Y, Yeh WW, Letvin NL, Mascola JR, Nabel GJ, Haynes BF, Bhattacharya T, Perelson AS, Korber BT, Hahn BH, Shaw GM 2009 Low-dose rectal inoculation of rhesus macaques by SIVsmE660 or SIVmac251 recapitulates human mucosal infection by HIV-1. J Exp Med 206:1117–1134. doi:10.1084/jem.20082831. 19414559 PMC2715022

[30] Ganusov VV, Auerbach J 2014 Mathematical modeling reveals kinetics of lymphocyte recirculation in the whole organism. PLoS Comput Biol 10:e1003586. doi:10.1371/journal.pcbi.1003586. 24830705 PMC4022467

[31] Miller CJ, Li Q, Abel K, Kim EY, Ma ZM, Wietgrefe S, La Franco-Scheuch L, Compton L, Duan L, Shore MD, Zupancic M, Busch M, Carlis J, Wolinsky S, Haase AT 2005 Propagation and dissemination of infection after vaginal transmission of simian immunodeficiency virus. J Virol 79:9217–9227. doi:10.1128/JVI.79.14.9217-9227.2005. 15994816 PMC1168785

[32] Hu J, Gardner MB, Miller CJ 2000 Simian immunodeficiency virus rapidly penetrates the cervicovaginal mucosa after intravaginal inoculation and infects intraepithelial dendritic cells. J Virol 74:6087–6095. doi:10.1128/JVI.74.13.6087-6095.2000. 10846092 PMC112107

[33] Couedel-Courteille A, Butor C, Juillard V, Guillet JG, Venet A 1999 Dissemination of SIV after rectal infection preferentially involves paracolic germinal centers. Virology 260:277–294. doi:10.1006/viro.1999.9809. 10417263

[34] Stone M, Keele BF, Ma ZM, Bailes E, Dutra J, Hahn BH, Shaw GM, Miller CJ 2010 A limited number of simian immunodeficiency virus (SIV) env variants are transmitted to rhesus macaques vaginally inoculated with SIVmac251. J Virol 84:7083–7095. doi:10.1128/JVI.00481-10. 20463069 PMC2898254

[35] Rothaeusler K, Ma ZM, Qureshi H, Carroll TD, Rourke T, McChesney MB, Miller CJ 2012 Antiviral antibodies and T cells are present in the foreskin of simian immunodeficiency virus-infected rhesus macaques. J Virol 86:7098–7106. doi:10.1128/JVI.00410-12. 22532691 PMC3416333

[36] Ma Z, Lu FX, Torten M, Miller CJ 2001 The number and distribution of immune cells in the cervicovaginal mucosa remain constant throughout the menstrual cycle of rhesus macaques. Clin Immunol 100:240–249. doi:10.1006/clim.2001.5058. 11465954

[37] Veazey RS, Marx PA, Lackner AA 2003 Vaginal CD4^+^ T cells express high levels of CCR5 and are rapidly depleted in simian immunodeficiency virus infection. J Infect Dis 187:769–776. doi:10.1086/368386. 12599050

[38] Veazey RS, DeMaria M, Chalifoux LV, Shvetz DE, Pauley DR, Knight HL, Rosenzweig M, Johnson RP, Desrosiers RC, Lackner AA 1998 Gastrointestinal tract as a major site of CD4^+^ T cell depletion and viral replication in SIV infection. Science 280:427–431. doi:10.1126/science.280.5362.427. 9545219

[39] Prodger JL, Gray R, Kigozi G, Nalugoda F, Galiwango R, Hirbod T, Wawer M, Hofer SO, Sewankambo N, Serwadda D, Kaul R 2012 Foreskin T-cell subsets differ substantially from blood with respect to HIV co-receptor expression, inflammatory profile, and memory status. Mucosal Immunol 5:121–128. doi:10.1038/mi.2011.56. 22089029 PMC3288185

[40] Balandya E, Miller AD, Beck M, Liu J, Li H, Borducchi E, Smith K, Cabral C, Stanley K, Maxfield LF, Barouch DH 2014 Adenovirus serotype 26 and 35 vectors induce simian immunodeficiency virus-specific T lymphocyte responses in foreskin in rhesus monkeys. J Virol 88:3756–3765. doi:10.1128/JVI.03771-13. 24429370 PMC3993544

[41] Dinh MH, Anderson MR, McRaven MD, Cianci GC, McCoombe SG, Kelley ZL, Gioia CJ, Fought AJ, Rademaker AW, Veazey RS, Hope TJ 2015 Visualization of HIV-1 interactions with penile and foreskin epithelia: clues for female-to-male HIV transmission. PLoS Pathog 11:e1004729. doi:10.1371/journal.ppat.1004729. 25748093 PMC4352059

[42] Ramskold D, Luo S, Wang YC, Li R, Deng Q, Faridani OR, Daniels GA, Khrebtukova I, Loring JF, Laurent LC, Schroth GP, Sandberg R 2012 Full-length mRNA-Seq from single-cell levels of RNA and individual circulating tumor cells. Nat Biotechnol 30:777–782. doi:10.1038/nbt.2282. 22820318 PMC3467340

[43] Schindler M, Munch J, Brenner M, Stahl-Hennig C, Skowronski J, Kirchhoff F 2004 Comprehensive analysis of Nef functions selected in simian immunodeficiency virus-infected macaques. J Virol 78:10588–10597. doi:10.1128/JVI.78.19.10588-10597.2004. 15367626 PMC516420

[44] Doitsh G, Cavrois M, Lassen KG, Zepeda O, Yang Z, Santiago ML, Hebbeler AM, Greene WC 2010 Abortive HIV infection mediates CD4 T cell depletion and inflammation in human lymphoid tissue. Cell 143:789–801. doi:10.1016/j.cell.2010.11.001. 21111238 PMC3026834

[45] Smith BA, Gartner S, Liu Y, Perelson AS, Stilianakis NI, Keele BF, Kerkering TM, Ferreira-Gonzalez A, Szakal AK, Tew JG, Burton GF 2001 Persistence of infectious HIV on follicular dendritic cells. J Immunol 166:690–696. doi:10.4049/jimmunol.166.1.690. 11123354

[46] Gonzalez SF, Lukacs-Kornek V, Kuligowski MP, Pitcher LA, Degn SE, Kim YA, Cloninger MJ, Martinez-Pomares L, Gordon S, Turley SJ, Carroll MC 2010 Capture of influenza by medullary dendritic cells via SIGN-R1 is essential for humoral immunity in draining lymph nodes. Nat Immunol 11:427–434. doi:10.1038/ni.1856. 20305659 PMC3424101

[47] Geijtenbeek TB, Kwon DS, Torensma R, van Vliet SJ, van Duijnhoven GC, Middel J, Cornelissen IL, Nottet HS, KewalRamani VN, Littman DR, Figdor CG, van Kooyk Y 2000 DC-SIGN, a dendritic cell-specific HIV-1-binding protein that enhances trans-infection of T cells. Cell 100:587–597. doi:10.1016/S0092-8674(00)80694-7. 10721995

[48] Miller CJ, McGhee JR, Gardner MB 1993 Mucosal immunity, HIV transmission, and AIDS. Lab Invest 68:129–145. 8441249

